# Towards an integrated approach in surveillance of vector-borne diseases in Europe

**DOI:** 10.1186/1756-3305-4-192

**Published:** 2011-10-03

**Authors:** Marieta Braks, Joke van der Giessen, Mirjam Kretzschmar, Wifrid van Pelt, Ernst-Jan Scholte, Chantal Reusken, Hervé Zeller, Wim van Bortel, Hein Sprong

**Affiliations:** 1 Laboratory for Zoonoses and Environmental Microbiology, National Institute for Public Health and Environment (RIVM), Antonie van Leeuwenhoeklaan 9, P.O. Box 1, Bilthoven, the Netherlands; 2 Laboratory of Epidemiology and Biostatistics, National Institute for Public Health and Environment (RIVM), Antonie van Leeuwenhoeklaan 9, P.O. Box 1, Bilthoven, the Netherlands; 3 National Centre for Monitoring of Vectors (CMV), New Food and Consumer Product Safety Authority (nVWA), Dutch Ministry of Economic Affairs, Agriculture and Innovation, Wageningen, the Netherlands; 4 Laboratory for Infectious Diseases and Screening, National Institute for Public Health and Environment (RIVM), Antonie van Leeuwenhoeklaan 9, P.O. Box 1, Bilthoven, the Netherlands; 5 European Centre for Disease Prevention and Control, Stockholm, Sweden; 6 Julius Centre for Health Sciences and Primary Care, University Medical Centre Utrecht, Heidelberglaan 100, 3584 CX Utrecht, The Netherlands

**Keywords:** Vector borne disease, surveillance, public health, ECDC

## Abstract

Vector borne disease (VBD) emergence is a complex and dynamic process. Interactions between multiple disciplines and responsible health and environmental authorities are often needed for an effective early warning, surveillance and control of vectors and the diseases they transmit. To fully appreciate this complexity, integrated knowledge about the human and the vector population is desirable. In the current paper, important parameters and terms of both public health and medical entomology are defined in order to establish a common language that facilitates collaboration between the two disciplines. Special focus is put on the different VBD contexts with respect to the current presence or absence of the disease, the pathogen and the vector in a given location. Depending on the context, whether a VBD is endemic or not, surveillance activities are required to assess disease burden or threat, respectively. Following a decision for action, surveillance activities continue to assess trends.

## Background

The European Centre for Disease Prevention and Control (ECDC) has a responsibility to identify, assess and communicate current and emerging dangers to human health from infectious diseases. Vector-borne diseases (VBDs) pose a special challenge to ECDC and national public health authorities due to their complex nature. Interactions between multiple disciplines and responsible health and environmental authorities are often needed for an effective early warning, surveillance and control of vectors and the diseases they transmit. Because many aspects of their complicated transmission cycle are under strong influence of environmental conditions (including weather), adequate risk assessment, early detection, prevention and control of endemic and emerging VBDs may demand approaches other than or in addition to those developed for non-VBDs. This was also recognized by ECDC and is their rationale for funding a network of both medical entomologists and public health experts, VBORNET. The network addresses the prerequisites for vector surveillance activities of arthropods of importance to priority diseases in the European Union. This paper aspires to bridge the gap between entomologists and public health professionals and to facilitate the communication between them. This first strategic paper of the VBORNET project aims to pinpoint the relevance of the different surveillance elements for VBDs important for public health in Europe. Because of the current focus of ECDC on tick-borne diseases, emphasis is placed on VBDs transmitted by ticks in Europe, in particular Lyme borreliosis.

### Definitions and basic concepts

#### Public health, medical entomology and vector-borne diseases

Conventionally, public health is defined as the branch of medicine concerned with the prevention and control of disease and disability in a population, and the promotion of physical and mental health of the population on the international, national, or intra-national administrative level. Currently, within the 'one health' initiative, however, medicine is considered more a branch of public health, due to the multidisciplinary character of the latter.

Medical entomology is the application and study of insect and other arthropod biology to disease transmission or sanitary matters. In addition to academic institutions, medical entomologists are commonly employed by vector control agencies, but only seldom by public and veterinary health institutes or governmental policymaking bodies.

In general, a vector-borne disease is one of which the causative agent is transmitted between vertebrate hosts by another organism (vector). Here, we use the definition in which an arthropod is required for the transmission and propagation of the pathogen. This narrow definition for vector-borne diseases is used because this class requires a common strategy for monitoring, surveillance and also control. In contrast, for pathogens that are optionally transmitted by vectors such as *Coxiella *or *Salmonella*, the approach for surveillance requires also other strategies related to other transmission routes.

From a public health point of view, five different types of VBD situations (contexts) are identified according to a simplified tabulation of the current presence or absence of the disease, the pathogen and the vector in a given location (Table 1). Here, a VBD is considered present when endemic infections in humans occur. In epidemiology, an infection is said to be "endemic" in a human population when that infection is maintained in the population without the need for external inputs (note that, in ecology, an organism being "endemic" means exclusively native to a place or biota). Furthermore, we consider a pathogen to be present, when it is circulating among indigenous vectors and non-human hosts, but also when it is regularly introduced by vectors or reservoirs, including humans. A vector is considered present when an arthropod capable of transmitting a certain VBD is indigenous. We elaborate on the different types of VBD contexts using the Netherlands as an example (Table 1).

**Table 1:** Different types of VBD context based on the current presence (√) or absence (-) of disease (endemic human cases), pathogen or vector, exemplified for the Netherlands

Context	Endemic Disease*	Pathogen	Vector	Examples of diseases holding for the Netherlands
1	√	√	√	Lyme borreliosis
2	-	√	√	Dirofilariasis
3	-	-	√	Tick-borne encephalitis
4	-	√	-	Leishmaniasis
5	-	-	-	Crimean-Congo haemorrhagic fever

* Endemic infections with human cases.

In the first VBD context, endemic human cases arise through the transmission of a pathogen to a susceptible human host from a reservoir host by an indigenous vector species. In the second VBD context, both pathogen and the vector are present but no human cases occur due to biotic, climatic, environmental or societal reasons. Such a situation can exist when a pathogen responsible for a zoonotic disease circulates in the animal reservoir and/or vector populations but without causing human disease burden, either because humans are not infected or because human infections go unnoticed. Low level circulation of West Nile virus in mosquitoes and birds in non-epidemic years in Italy and France are other examples [1,2]. In the third VBD context, a competent vector is present but the pathogen has never been introduced and therefore there are no locally transmitted human cases. The reverse circumstance is described in the fourth VBD context where a pathogen is frequently imported into a location where no competent vector is present, which precludes pathogen transmission and therefore autochthonous human cases. The frequent importation of *Leishmania*-infected dogs from the Mediterranean countries to the Netherlands is an example of the latter. Locations where both the vector and pathogen are absent fall into the fifth type of VBD contexts. Despite incidental findings of a *Hyalomma *tick, the vector of Crimean-Congo haemorrhagic fever (CCHF) [3], the situation of the Netherlands with respect to CCHF is of the latter type.

In short, all endemic VBDs fall under context 1 and the various non-endemic VBDs fall under one of the remaining four contexts (2-5). To identify, assess, communicate and ultimately control VBDs, monitoring and surveillance tools, appropriate to the context, are needed.

#### VBD surveillance feedback system

According to ECDC, *"Surveillance of health and disease includes ongoing data collection, analysis to convert this data into statistics, interpretation of this analysis to produce information and dissemination of this information to those who can take appropriate action. At ECDC, this process is aimed at providing appropriate, quality and timely information for key stakeholders in Member States and European Commission. This permits them to take action by planning and implementing more effective, evidence-based public health policies and strategies relevant to the prevention and control of communicable disease in crisis situations as well as in the long term" *(ECDC, 2011).

**Figure 1:**
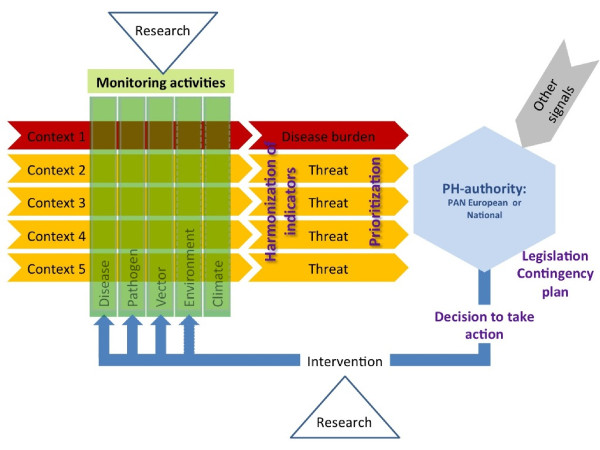
**Simplified scheme for VBD surveillance feedback system**. Vertical yellow cylinders represent the categorical collections of data (monitoring). Horizontal arrows represent the use of selective set of data from monitoring activities for surveillance purposes for a specific VBD context or VBD. The diamonds points at the responsible health agency/decision makers and the triangles represent the input of research and developmental studies. The EU could play a pro-active role in the activities indicated in purple. Other signals indicate signals that are not included in disease burden or threat assessments but that affect decision-making.

Generally, estimations of the current impact of a health problem are made through calculations of the burden of disease and the assessments of the impact of future outbreaks in an area through quantitative microbial risk assessments. While applicable for endemic VBDs (context 1, Table 1, Figure 1), such quantitative assessments would not be sensible for the remaining VBDs contexts with no actual disease burden or risk (the latter defined as probability times impact). Nevertheless information on these so-called threats to human health is desirable.

Essential for the assessments of both the burden and the health threat is the identification of the appropriate indicators defined as measurable factors that allow decision makers to estimate objectively the size of a health problem (disease burden) or severity of a threat. These indicators should be appropriate for monitoring the processes or the effects of intervention aiming at decreasing the disease burden (VBD context 1) or preventing the introduction of disease burden (VBD contexts 2-5). For VBDs, indicators not only relate to occurrence of human disease, but also on the abundance of pathogen and/or vector (see Table 1, Figure 1). In addition to identification of the adequate indicators, harmonization (and communication) of these indicators across diseases and countries would also be very advantageous.

Priority setting of communicable diseases has been attempted in several countries. Disability-adjusted life years (DALY) [4] are often used for a standardized quantification of the disease burden. The DALY approach combines mortality, incidence (and/or prevalence) of infection and the sequelae associated with an infection on the population level into a single measure [5-7]. Policy-makers use this indicator for setting priorities in their decision-making. In order to obtain DALY estimates, monitoring of disease indicators is required in each country and demands a lot of effort. Data sets are (still) not comparable across EU-countries, but efforts are ongoing to develop and estimate disease burdens of communicable diseases in Europe [8]. For non-endemic infectious diseases (VBD contexts 2-5), however, the priority setting process is more complex as there are no endemic cases. Often an emerging infection is only a threat and data on essential criteria for priority setting for national or even the European situation may not be available as there is no endemic or epidemic disease burden. To quantify the threat of an emerging infection, DALY's per expected incident case need to be estimated to assess the possible impact of an outbreak. Recently, approaches of priority setting of emerging infectious diseases, including VBDs, were described based upon a set of criteria and expert opinions, where possible including data from the available literature (http://www.ezips.rivm.nl; [9,10]). Other priority-setting criteria for assessing the threat of emerging pathogens entail their potential to spread among the general population, their associated socio-economic burden, their preventability, their potential to drive public health policy, their perceived risk for public health, their temporal changes in occurrence and their perceived potential to cause an outbreak.

Decision making, whether to intervene or not, needs to follow the priority setting process taking cost-benefit ratio into account. Especially, in a time of grim governmental budget cuts, focusing on interventions that achieve the largest health gain per euro spent is sensible. For some VBDs, taking action even when as yet there is no disease, might be preferable.

Once a decision to intervene has been made to decrease the disease burden (or group/category of diseases) or to mitigate a threat, surveillance should be implemented in order to measure the effectiveness of the intervention.

Within the VBD surveillance feedback system three main situations can be distinguished:

1. The collected information does not directly provide estimations of disease burden or threat. Consequently, they do not trigger decisions whether to take action or not, but are merely carried out to investigate unknowns of a VBDs transmission, ecology and/or epidemiology, e.g. tick densities or proportion infected ticks.

2. The information enables the estimation of disease burden or threat. However, based on the provided data, the decision was made that intervention is not opportune. Surveillance activities continue to follow trends in the situation.

3. The provided information on disease burden or threat leads to the decision to intervene. Control actions are developed and implemented. Surveillance activities continue in order to measure the effectiveness of the intervention on reducing disease burden or threat. To do so, surveillance activities also assess change in the direct intervention parameters. For example, in a situation where intervention is implemented to lower the disease burden of Lyme borreliosis by controlling ticks, the change in tick numbers is assessed, and not only the change in disease burden.

Obviously, decision making is a crucial step in VBD surveillance feedback system.

#### Data collection

Data collection is either passive or active. In veterinary and public health systems, passive activities involve voluntary or mandatory reporting of cases, either animal or humans, that are ill enough to have consulted a practitioner. Active systems involve 'searching' for evidence of disease through routine, periodic or continuous data collection [11]. Active surveillance provides the most accurate and timely information but is also expensive [12]. Passive activities are less expensive, but because only infected subjects with clinical signs are included, they do not provide complete information about the circulation of a pathogen in the population (true prevalence).

The following five types of data collection can be distinguished with respect to the parameter that is considered, exemplified by issues concerning VBDs:

1. Pathogen data collection that focuses on pathogen detection and identification of all levels, including human case, reservoir host, and intermediate host, but also within the vector.

2. Serological data collection that aims at the detection of exposure to a pathogen by monitoring immunological responses in the blood of animals or humans.

3. Clinical data collection refers to the monitoring of a clinical syndrome that has a significant impact on veterinary or public health, which is then used to drive decisions about health policy and health education. In human health this predominantly refers to passive surveillance i.e. making use of data collected from patients for other purposes than surveillance (e.g. declaration, patient dossier or management). If additional data are collected from the patient explicitly for public health surveillance, it is called active or intensified surveillance. Parallel collection of data from controls may be part of the design in active surveillance allowing continuous periodical case-control analysis. In that case the distinction with classical epidemiological case-control studies is that data collection is not restricted to a predefined time period.

4. Syndromic data collection refers to an active or passive system that uses case definitions that are based entirely on clinical symptoms without any differential or laboratory diagnosis.

5. Risk data collection that focuses not on prevalence of pathogens or clinical features in animals or humans, but on detecting risk factors for disease transmission. The collection of data on the presence/absence and abundance of vectors falls in this category. In addition, it can also include behavioural risk factor surveillance [12], which involves the active system of repeated surveys that measure behaviours that are known to cause infection (e.g. exposure to infected ticks). The presence and activity of vectors are susceptible to weather (climate) and environmental conditions (flora, fauna, and landscape design). These data also fall into this category. Especially the presence of vectors can be obtained both actively and passively (see section on parameter needs for vector-borne disease monitoring in Europe).

The use of standards or the harmonization of data collection, representation and reporting facilitates comparability of information over time, across different approaches and across countries and regions, and allows integration of collected data sets for more powerful analyses. To be credible, a standard should be developed through open participatory process by an internationally recognized accredited standards development organization that is also capable of long-term maintenance and evolution of standards [12].

#### Early warning and preparedness

Early warning is a system in which reception of certain predefined signals will trigger interventions. For example, upon the introduction of a certain exotic vector species (a list of important invasive vector species should be available), vector control measures will be taken. In endemic situations an early warning is triggered when the frequency of occurrence of a disease crosses a predefined threshold [13].

Preparedness is a system in which scenarios are described and public health authorities are prepared for outbreaks of diseases prior to their arrival (see also Figure 1). In the case of an introduction of a certain pathogen, the authorities should have information available on whether a vector is present or not. One of the consequences of preparedness can be, for instance, that when no competent vector is present (VBD context 4), no further actions will be taken to prevent the pathogen from spreading in the country. However, the existing knowledge must be carefully assessed before taking any decision, especially considering the low level of understanding of the vectors' transmission competence. A more complicated situation can occur where action might be needed, for instance when a sudden introduction of a new vector species occurs, as was the case recently with the introduction of a mosquito species imported with used tyres [14,15]. In this case immediate action might be required, but at the same time, a contingency plan, legal requirements and responsibilities (for example on usage of pesticides) should already be in place.

### Parameter needs for vector-borne disease monitoring in Europe

#### Disease burden

**Figure 2:**
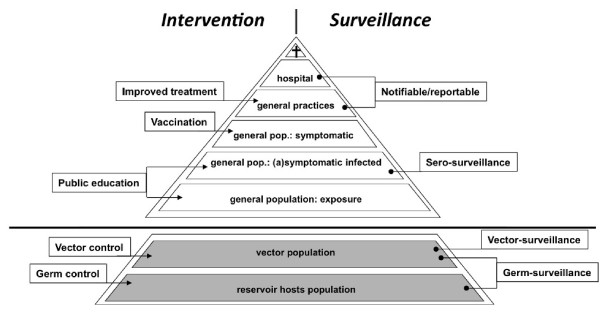
**Intervention and surveillance pyramid for vector-borne diseases**.

To decide which VBDs should preferably be controlled, we need estimates of the actual disease burden for endemic infections. To adequately monitor the disease burden of VBDs related to their occurrence, information is needed from different subgroups of the human population, represented by a disease burden pyramid (see also Figure 2). Patients, who are hospitalized or who died form the top of this pyramid for which different monitoring systems are in place. The following layer contains all laboratory confirmed cases, which are reported to the surveillance system of a local or state health department. Periodic epidemiological studies in the general population are necessary to assess the total number of diseased persons and the proportion that presents to the health care system (layer 3 from the top of pyramid, Figure 2) [16]. Ideally such a system for VBDs should involve laboratory surveys, physician surveys, and population surveys to collect information about each of these steps. An additional layer in this pyramid about asymptomatic and symptomatic cases estimated by serology [17] is necessary for disease burden estimations.

Gathering data for the estimation of the disease burden has been quite successful for several food-borne infections [5]. However, hardly any data exist on the basis of which the disease burden of VBDs can be estimated. Alternatively, parameters that are indirect indicators, so called proxies, of disease burden are used and monitored. In quantitative microbiological risk assessments, for example, data about those exposed but not necessarily infected, is primarily needed. Information about exposure to VBD agents depends on contact patterns between humans and infected vectors. These differ between mosquito-borne, tick-borne, and sandfly-borne diseases, but also within these three categories. Monitoring of contacts between humans and vectors requires information about habitats of vectors, behaviour and seasonality of vectors, and human behaviour that enables the vector to bite or feed on humans. Human behaviour can be monitored in population surveys or by defined specific target groups with possibly high risks of exposure (sentinel populations). Possibly, web-based monitoring of human behaviour offers an opportunity for monitoring trends in risk behaviour. The usefulness of extending internet applications as for monitoring influenza (http://www.degrotegriepmeting.nl/) to VBDs should be investigated. Serological surveys can give insight into changes in exposure by estimating the number of seroconversions, possibly both past and recent. Large population based sero-surveys used for other purposes (e.g. effectiveness of a national vaccination program [18]) could be applied to test for antibodies against vector-borne infections. Sero-surveys could be accompanied by questionnaires concurrently asking for bites and pathognomonic symptoms (e.g. erythema migrans (EM) in case of Lyme borreliosis) in the past five years. Trends in exposure similar to that of the results of laboratory surveillance can be used to update earlier disease burden estimates. The occurrence of (self-reported) symptomatic infections (in case these are pathognomonic for a certain disease), bites and care-seeking, can be monitored prospectively via web-based systems of questionnaires in large predefined cohorts for a series of VBDs as done for other series of health care questions (http://www.degrotegriepmeting.nl/). Taken together such approaches may allow an estimate of the number of symptomatic VBD infections [16] and care seeking in the general population. A certain fraction of symptomatic infections will be tested and diagnosed by a general practitioner (GP). For these infections, a GP sentinel system can monitor trends in testing and positivity. Comparing the testing rate with the rate of self-reported symptomatic infections gives an estimate of the fraction of infections that lead to a GP visit and the fraction that remains untreated.

Hospital referral and sequelae to infections from VBDs could be obtained from GP sentinel systems as well in addition to data from hospital registration systems. Mortality due to VBD, in principle, can be obtained from mortality monitoring by cause of death.

### Disease burden of Lyme borreliosis: An example

**Figure 3:**
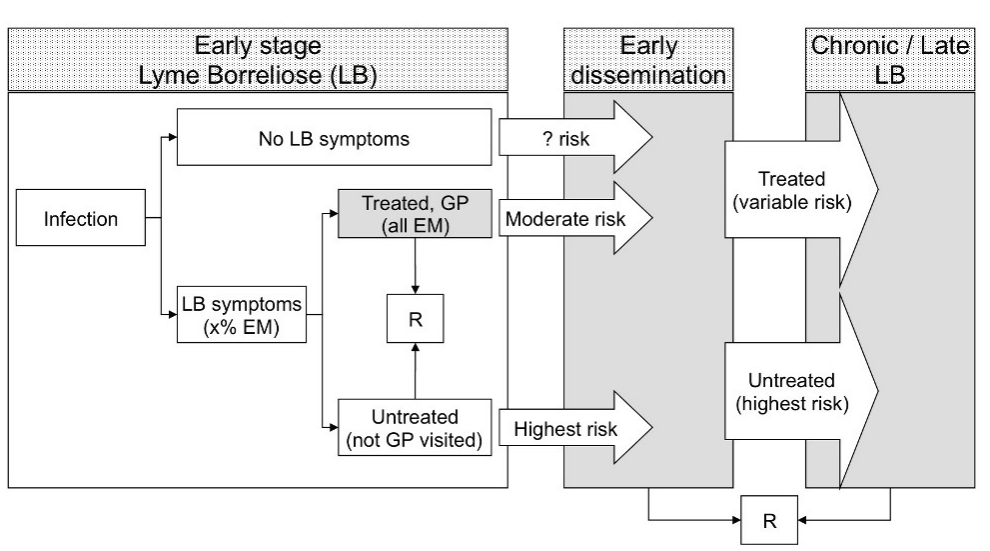
**Outcome tree for estimating disease burden of Lyme borreliosis (LB)**. R: recovered. GP: general practitioner. An outcome tree for estimating economic costs of LB is presented in [21].

Although it is acknowledged that Lyme borreliosis is increasing in many countries [19,20], the extent of the problem is difficult to communicate to policy makers. For Lyme borreliosis, attempts to estimate the disease burden have been undertaken using an outcome tree (Figure 3) and information about the incidence, cost of illness and burden of each of the sequelae of the infection [21].

Due to the lack of the pathognomonic features of the clinical symptoms with the exception of erythrema migrans, and the very low sensitivity of serological tests, incidence rates needed for estimating the disease burden may be largely underestimated or uncertain. The lack of evidence for Lyme borreliosis such as no tick noticed, no erythrema migrans no positive serology will lead to underdiagnosis and underreporting of the sequelae due to the infection.

**Figure 4:**
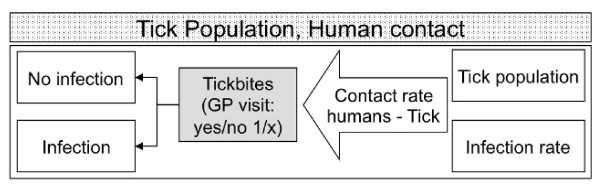
**Outcome tree of human exposure to ticks**. ^†^total tick bites is GP visits for tick bites times 15 (x) in the Netherlands [23].

The number of tick bites relates to the entomological risk index and depends on the tick population and contact rate of humans and ticks (Figure 4). Exposure to ticks in itself may lead to seeking medical care [22]. In the Netherlands, monitoring GP consultations for tick bites concurrently with answers from questionnaires in the general population on tick bites [23] shows that one out of fifteen tick bites leads to visiting a GP. Accurate numbers of patients with consultations for EM exist as well for the Netherlands [19,23]. Almost all of these patients are expected to be treated according to national medical guidelines. However, therapy failure in 10-20% of cases is estimated in the literature [24,25]. Moreover, at least an equal number of eythrema migrans patients do not visit their GP and will only be treated after they develop late-stage sequelae. Not all infections have an early manifestation with an erythrema migrans, a range from 75-90% with erythrema migrans is reported [26]. Anecdotal evidence suggests that depending on the *Borrelia *spp. this might even be as low as 50% or lower. Clearly, getting the figures correct is a major challenge warranting a concerted action on an international level.

In the Netherlands, methods measuring proxies of disease burden for Lyme disease are under development. In 2011, all physicians are approached to provide retrospective data and to forward extensive questionnaires to their LB patients in the past 12 months. Patient questionnaires are used to reconstruct the evolution of their disease, at what stage they started seeking medical care, therapy failure, related costs and quality of life.

### Parameter needs for vector and pathogen monitoring in Europe

#### Threat

The data collection described above generally applies for assessing disease burden of an endemic pathogen. However, the forecasting, early detection, prevention and control of emerging VBDs assess threat (Table 1, context 2-5) demand knowledge of other parameters. Acquiring data on the density of (infected) vectors complements to the disease burden pyramid for VBDs (Figure 2). In case of a zoonotic VBD, yet another level is added, that of the number of (infected) reservoir hosts (Figure 2). Infection rates of vectors are indicators of risk of transmission. Sustained elevated infection rates (assuming baseline data are available) are indicative for an increased transmission risk. Combining the infection rates with vector abundance (i.e. density/activity) will result in a more accurate estimate of the risk of infection. An integrative approach combining the modelling of the reproduction numbers of VBD and the construction of risk maps to predict the emergence of VBD was described recently by Hartemink [27].

Vector sampling programs are essential to determine presence/absence and abundance of vectors for risk surveillance and preparedness, but also for pathogen surveillance at the vector level. For the latter, it is important to be able to distinguish between the different levels of pathogenicity (pathotypes) of particular pathogens, or at least between non-pathogenic and pathogenic species. Since VBDs, by definition, require vectors for their transmission, the absence of vectors put the transmission of VBDs to a halt. As a consequence, eradication or control of the vector population is an important intervention tool for VBDs. To measure the effect of vector control, indicators on the presence/absence and abundance of the vector in a given location are essential. In addition, the longevity of individual vectors is also an important determinant, but difficult to monitor. The control of the major host of the vector or the reservoir host(s) of the pathogen may also be means to control the disease.

#### Presence/absence and abundance data of vectors

While data on the size of the study population (and consequently the sampling fraction) are readily available for domestic animals and humans, basic information on vector distribution and abundance does not exist for most countries or locations. The most important epidemiological parameters for vector monitoring are vector diversity (presence/absence of species), spatial and temporal variation in vector abundance and pathogen prevalence in the vector population. These parameters can usually only be gathered through active vector surveillances and pathogen prevalence assessment in the collected vectors. In contrast to biases in passive surveillance, sampling strategies in active surveillance can be largely kept under control. Active surveillance, however, is also more costly and labour intensive than passive surveillance. In specific cases, involving mostly ectoparasites such as ticks, passive surveillance alone gives very useful information and is even preferred [28]. Sightings of species by the public or volunteers can aid species diversity databases, but cannot be used for spatio-temporal species mapping due to large biases. Since arthropod species diagnostics is such a specialised area of expertise, specimens that are collected by the public, should not be included in such data-bases without verification by an expert. The down-side of passive surveillance, generally, is that it comprises only presence data of species (specimen) and not data showing the absence of species. Absence data are just as important as presence data. But if no specimen of a species is collected in an area, it does not prove that that species is absent from that area; it might be rare, hiding, be in an inactive state, be in a different type of habitat, or not be responding to the trapping device used. In conclusion, although absence data are generally more difficult, costly and labour intensive to gather, they are indispensable for surveillance of vector-borne diseases. Making presence/absence maps of important vector species is essential for determining to which category the VBD in a certain location belongs, to VBD contexts 1-3 or VBD contexts 4-5 (Table 1). In the first case (VBD contexts 1-3) vector surveillance can entail measuring the spatial and temporal variation in vector abundance and pathogen prevalence.

Presence/absence data sets would be ideally derived from databases of local monitoring programs assessing the spatial and temporal abundance patterns of vectors, which may be obtained by systematic methods involving consistent sampling and standardized collection protocols. However, more often data is assembled from research projects addressing other questions than presence/absence, or from control agencies. For arthropod vectors such methods typically produce data expressed per unit such as the number of adult mosquitoes per trap night, or effort such as the number of nymphal ticks per flagging hour or drag-meters. These measures are relative numbers and do not estimate density (number per unit area) or absolute size (total number as measured in mark-release-recapture methods). Accuracy is important as thresholds of abundance such as counts per night are used to make decisions on the density of the vector-borne health risk or the type of focus and intensity of control. Precision and standardization are important if estimates are compared over time and or space. A sampling program consists of a program design, data collection and statistical analyses. However the data analysis is only as informative as the validity of the samples. Studies specifically designed for the collection of absence/presence data would be highly desirable.

Since scientific evidence has established the trend of ongoing climate change related to human activities, the potential for climate-induced change in distribution patterns of arthropod vectors and their associated pathogens has emerged as an area of special concern to human and animal health. Model simulations of potential future climate related change in spatial patterns of distribution or abundance of vectors, distribution or prevalence of vector-borne disease agents in vector populations, or distribution or incidence of vector-borne diseases affecting humans or domestic animals have been presented [29]. However, when the possible effects of climate changes on specific vector-borne diseases are analysed in detail, the results are often inconclusive and the strength of a potential link weak. In the case of tick-borne diseases, such as tick-borne encephalitis, climate is considered just one of many factors, biological and non-biological that influence tick-borne diseases dynamics [30]. Future empirical demonstrations of climate-driven change in spatial and temporal patterns of ticks and their associated pathogens are entirely dependent on the existence of high-quality field data sets for current patterns in areas where climate change is likely to have a significant impact on the vector presence and abundance [29].

#### Pathogen prevalence in vectors

In theory, vector-borne pathogens can be detected in donor or recipient individuals (both human and animal), and in the vector. For monitoring purposes, detection of pathogens in vectors is often the method of choice as vectors might accumulate different pathogenic species from various different sources of reservoir hosts and the presence of the pathogen in vertebrate host can be sometimes of short duration (e.g. for arboviruses). Pathogen detection in wildlife is very laborious and often not possible because of the protected status of many wild animals that precludes the sampling from target organs as is often required for detection. Companion animals such as dogs and horses have been used (successfully) as sentinel animals for human infection [31]. Pathogen detection in vectors collected from humans give a direct insight in public health relevant vector species and might also be used for studying transmission risks. Additional information can be acquired from ticks collected from livestock, and companion animals. Blood-meal analyses in vectors, in combination with pathogen detection, may be a powerful tool to determine the relative importance of hosts to the vector population, and also the distribution and prevalence of pathogens in feeding hosts.

One of the major concerns in conducting monitoring programs to detect pathogens (especially in case of viruses) in vectors is the handling of field-collected mosquitoes and ticks. In case of arbovirus detection, RNA degradation should be prevented, and vectors should be maintained in a cold chain through the various handling procedures or immediately after killing be submerged in RNA preservative buffer to reduce the risk of pathogen inactivation [32-34].

Traditional circumstances in the field-collection might not allow for the maintenance of a cold-chain or the prompt processing of the mosquitoes (fresh kill) upon capture. The use of a RNA preservative might increase RNA stability under adverse conditions but is not recommended when species identification is required [35]. The goals of the surveillance need to be considered when determining the handling conditions that should be employed. Is there a need for isolation of infectious virus or does the simple establishment of the presence of a specific pathogen in mosquitoes suffice? The maintenance of a cold chain is absolutely required when infectious virus needs to be isolated [36]. An important advantage of infectious virus assays is that a wide variety of pathogens can be detected while RT-PCR assays only detect those pathogens and strain variants that the primers are designed to detect. This is in particular important for (high risk) areas where a variety of arboviruses might circulate. Employing RT-PCR detection might lead to failure to detect viruses that are not suspected to circulate. Especially in low endemic areas, it is critical to implement a quality check of the RNA isolated from the arthropods to avoid false negative results due to the failure to extract good quality RNA. Several strategies to implement an internal positive control for RNA isolation in mosquitoes have been described [37,38].

For some pathogens the infection rate is extremely low, even in highly endemic areas. One option is to analyse a number of specimens (minimally separated by species) together in one pool. In general, increasing the pool size will decrease the test sensitivity. In high endemic areas a loss in sensitivity is more acceptable than in low endemic and in particular arbovirus-free areas. On the other hand the total number of mosquitoes that need to be analysed (and therewith the practical need to increase pool sizes) will be considerably higher in low endemic and arbovirus-free areas. The extent of the sensitivity loss with increasing pool sizes will depend on the virus-mosquito species combination and the detection assay used (Reusken, Pers. comm.) [39]. Also, increasing the pool sizes will reduce the ability to estimate accurately the proportion of infected specimens, especially when proportions are high during an epidemic or sample sizes are low [40,41]. Infection rates are important indicators for virus transmission risks although low infection rates do not exclude transmission risks. Furthermore, increasing pool sizes will increase the possibility that more than one virus is present in the sample (e.g. in regions endemic to multiple VBDs), complicating confirmation of virus or virus strain identification by sequence analysis. The generalized procedure for arbovirus surveillance in mosquitoes is the processing of pools with a maximum of 50 individual specimens [32,39,42-44].

Microscopic analyses of vector-lysates have been gradually replaced by PCR-based techniques. PCR-based techniques greatly improved sensitivity, specificity, and throughput capacity of the number of samples and number of pathogens to be tested. However, PCR-based technologies have some limitations, which should be taken into account during surveillance activities.

PCR-based methods are based on the detection of specific DNA sequences: It does not necessarily mean that viable, infectious microorganisms are detected! PCR may result in the amplification of a wrong target DNA or RNA. For the purpose of early warning, the proportion of false positive test results should be minimized [45,46]. In that case, confirmation of test results and thus minimizing false-positive results by further specification can be done by using a DNA hybridization probe, which is often used in Q-PCR and array-based methods [47], or by sequencing the PCR product and comparing to a reference database. For pathogen identification, Genbank is often used but should only be done with caution because of many errors. Although extremely specific, misidentification may still occur, especially when relatively short or highly conserved DNA/RNA-regions are being used. For surveillance purposes, sequencing is still a laborious, slow and relative expensive technique, but second and third generation sequencing approaches may overcome these disadvantages. PCR is also not stage specific (e.g. malaria). Another obvious disadvantage of PCR is that it identifies the presence of DNA/RNA with high sensitivity. PCR may detect (remnants of) microorganisms present in the blood meal, which are inactivated, dead and non-infectious to the next host. A consequence is that PCR can also be used to determine the previous host of the vector [48]. Other examples are the finding of *Borrelia henselae, Rickettsia typhii *and *R. prowazeki *in questing ticks [49,50]. Besides aspecific binding, a PCR may fail to detect a pathogen, simply because it is present below the detection limit of the PCR, or because of the presence of inhibitory factors, such as haemoglobin or chitin. Highly specific primers may also fail to bind because of the high genetic variability of the target DNA/RNA. Either using another or multiple target DNA/RNA fragments of the pathogen of choice can circumvent this problem.

### Surveillance and intervention

Surveillance of vector-borne diseases aims at the accurate and timely measurement of the introduction of vectors and pathogens, the incidence of disease, and the effect of preventive, control and curative actions taken.

The surveillance feedback system strongly depends on the actions that will be taken upon the outcomes of the surveillance. For example, the control option for measles and mumps is vaccination. A surveillance system measuring newly reported cases of measles and mumps as the indicator parameter is sufficient. In situations where more intervention measures need to be considered or are taken, the identification of indicators that measure the effect of intervention is more challenging. An intervention, for example, that aims to raise the awareness of the risk of acquiring Lyme borreliosis through tick bites might increase the number of reported tick bites or even erythema migrans, but the number of Lyme patients with chronic or late-stage symptoms would decrease. Moreover, the best indicator for an intervention involving tick control on large mammals would be the number of ticks on large mammals. However, acquiring such data is very costly. An alternative indirect indicator here would be the tick density in the environment. Obviously, in a feedback VBD surveillance system (Figure 1), the parameter measured in monitoring of VBDs, vector and pathogen can often also be used to assess the effectiveness of intervention. The integration of intervention and surveillance system is illustrated in Figure 4.

In case the infection is present at an endemic level (VBD context 1, Table 1) e.g. Lyme borreliosis in the Netherlands, the indicator requirements of a VBD surveillance feedback system in the human population resemble those of other notifiable diseases with the exception of exposure surveillance, which depends on the human - vector contact patterns. The disease burden pyramid, complemented with the two additional layers, becomes suitable for the surveillance system for VBD and probably all zoonotic diseases.

### Decision making

In the preceding paragraphs, we have argued that the key objectives of VBD surveillance feedback system are to understand the epidemiology of VBDs and to provide adequate information on risk-assessment for decision makers to decide by disease burden or threat calls for intervention measures. To this end, local, national and international (health) authorities need to know in which situation they find themselves for a given VBD with two main groups: endemic (Table 1, context 1) or non-endemic (Table 1, context 2-5). For many VBDs, exemplified with Lyme borreliosis, disease burden assessments are fraught with difficulties. Measurements of indirect indicators of disease burden with information from the base levels of the pyramid have proven to be useful [23]. Standardisation and harmonization of indicators of disease burden or threat are desirable.

When intervention is decided to be desirable, a different approach is needed for a VBD belonging to one or the other main group. Intervention of endemic diseases can be implemented on either level of the pyramid, depending on the pathogen and/or vector of interest. Intervention, however, of a non-endemic VBD should be implemented on the two base levels of the pyramid. Once a decision has been made to control a certain disease (or group/category of diseases) or threat, surveillance should be conducted in order to measure the effectiveness of the intervention.

## Conclusions

This paper intended to improve the mutual understanding and enhance communication between the communities of medical entomology and public health. Both worlds need to work together to assess the disease burden or threat of vector-borne diseases and to decide on intervention measures. To this end, we developed a framework for a surveillance feedback system for VBDs, which share many characteristics with other infectious diseases. However, because of the involvement of a vector (and non-human reservoirs, in the case of vector-borne zoonoses), additional issues need to be considered, as illustrated by the complementary layers to the disease burden pyramid, together forming the intervention and surveillance pyramid for VBDs.

For surveillance purposes, international, national and local (health) authorities need to know in which situation they find themselves for a given VBD (table 1). We suggest that countries consider implementing a dynamic database in which this information is established and kept up to date. This information can then also be reported to ECDC. ECDC might facilitate this by providing an international framework/platform comprising (molecular) epidemiological databases on vectors, pathogens, and disease, which is useful for the collection, sharing and interpretation of the data at both the national and international level. A clear and qualitative insight in the current situation will then be available for risk assessment.

Besides data on the presence of diseases, pathogens or vectors, convincing data for true absence is essential. Evidence for the latter is, however, much harder to provide. Furthermore, insights from recent scientific and medical developments should be incorporated into the list in Table 1: (Potential) new vectors and vector-borne pathogens are reported in the scientific literature frequently, including vector competence. Input from research and developmental studies are absolute requirements to keep the information of Table 1 up to date, both at the national and international level.

The availability of information, however, to assess the context of a certain disease situation should be based on monitoring data, for which availability differs greatly between regions or countries. Pan-European surveillance activity could be instrumental for ECDC and their member states to prioritize their future activities, based on evidence. When deemed necessary, contingency plans, including intervention strategies, can be developed and surveillance activities implemented as integrated part of the VBD surveillance feedback system guiding the Member States. Interactions between multiple disciplines and responsible ministries are essential for an effective and efficient early warning, surveillance and control of vectors and VBDs.

We hope to increase the awareness of VBD situations in EU, and to present a first step for decisions on the implementation of more harmonized surveillance activities by EU member and associate states.

## Competing interests

The authors declare that they have no competing interests.

## Authors' contributions

All authors participated in the formation of the manuscript's content. MB coordinated the preparation and writing of the manuscript. All authors contributed to helpful discussions, read and approved the final manuscript.
